# Cell therapies for Parkinson's disease: how far have we come?

**DOI:** 10.2217/rme-2016-0102

**Published:** 2016-11-25

**Authors:** Thomas B Stoker, Roger A Barker

**Affiliations:** 1 John van Geest Centre for Brain Repair, Department of Clinical Neurosciences, University of Cambridge, Forvie Site, Cambridge, CB2 0PY, UK; 2 Wellcome Trust – Medical Research Council Stem Cell Institute, University of Cambridge

**Keywords:** cell-based therapies, embryonic stem cells, fetal ventral mesencephalic tissue, induced pluripotent stem cells, neural grafting, Parkinson's disease

## Abstract

Over the past three decades, significant progress has been made in the development of potential regenerative cell-based therapies for neurodegenerative disease, with most success being seen in Parkinson's disease. Cell-based therapies face many challenges including ethical considerations, potential for immune-mediated rejection with allogeneic and xenogeneic tissue, pathological spread of protein-related disease into the grafted tissue as well as the risk of graft overgrowth and tumorigenesis in stem cell-derived transplants. Preclinical trials have looked at many tissue types of which the most successful to date have been those using fetal ventral mesencephalon grafts, which led to clinical trials, which have shown that in some cases they can work very well. With important proof-of-concept derived from these studies, there is now much interest in how dopaminergic neurons derived from stem cell sources could be used to develop cell-based therapies suitable for clinical use, with clinical trials poised to enter the clinic in the next couple of years.

**Figure 1.**
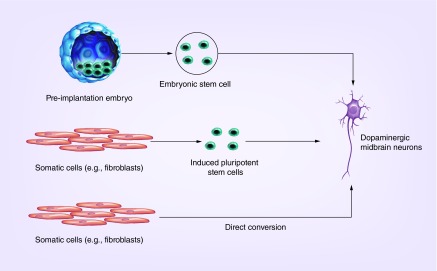
**Potential sources of cell-based therapies for Parkinson's disease.** Authentic midbrain dopaminergic neurons may be generated from embryonic stem cells harvested from preimplantation embryos induced pluripotent stem cells derived from adult somatic cells, and directly from adult somatic cells (induced neurons).

Many chronic neurodegenerative conditions are characterized by the degeneration of a specific population of neurons, such as the dopaminergic neurons of the nigrostriatal pathway in Parkinson's disease (PD), the striatal medium spiny neurons in Huntington's disease or the anterior horn cells in motor neuron disease. As such, there has been much interest in the development of cell-based therapies to replace deficient neuronal pathways, with the first experiments of grafting cells into the brain occurring in the late 19th century [1]. However, it is only in the last three decades that significant developments in this field have been made, and with this the prospect of clinically useful therapies has emerged.

Neural grafting has been trialed in several neurodegenerative conditions but progress has been greatest in the field of PD, which will be the main focus of this review. The motor manifestations of PD can be treated with dopaminergic medications, but over time these lead to significant side effects including levodopa-induced dyskinesias and neuropsychiatric manifestations, secondary to the nonphysiological release of dopamine and activity at dopaminergic pathways other than the nigrostriatal pathway [2,3]. These effects contribute significantly to the morbidity associated with advancing PD, and as such a more physiological means of delivering targeted dopamine to the basal ganglia are needed, and one such way would be to use transplants of dopaminergic neurons. In this review we will therefore concentrate on the evolution of cell-based therapies in PD, which aim to fulfill this need, as well as discussing the challenges of using this approach.

## Challenges for cell-based therapies

Progress in the development of regenerative cell-based therapies for neurodegenerative conditions has taken several decades, partly due to inherent technical challenges in establishing the optimal approaches, but also due to unique challenges that are not seen with more conventional treatments in neurological disease. Below we discuss different sources of cells for potential transplantation (Box 1), but those involving fetal or embryonic tissue particularly bring about important ethical considerations [4]. Immune-mediated rejection of grafted tissue is another barrier that must be overcome, and the optimal immunosuppression regime must be determined to allow graft survival and longevity. Inadequate immunosuppression regimes may have contributed to the modest results seen in some of the trials of human fetal ventral mesencephalon (fVM) grafts for PD, which are discussed below [5,6]. Finally as we move toward more stem cell-based therapies, the potential for graft overgrowth, or development of tumors secondary to transformation events in the grafted cells needs to be considered along with the abnormal migration of cells out of the transplant.

Although clinical manifestations of some conditions occur due to loss of a specific subtype of neurons, for most neurodegenerative diseases it is an oversimplification to think that replacement of a specific cell type will reverse all of the effects of the disease and this includes PD. In this condition it is known that areas other than the dopaminergic neurons of the substantia nigra are involved in the disease process and therefore any dopamine cell-based transplant will only ever treat limited, albeit important, aspects of the condition. Finally, another disease-related challenge facing cell-based treatments is the fact that disease may recur in the grafted neurons. For example, Lewy body pathology has been identified at post-mortem in patients that received fVM grafts many years ago for their PD [7,8].

## Preclinical studies

Neural grafting in animal models initially provided important proof-of-concept data, demonstrating that cell-based therapies could reverse some of the clinical manifestations of specific lesions. The 6-hydroxydopamine-lesioned rat model for PD has allowed for the investigation of restoration of dopaminergic activity in a dysfunctional nigrostriatal pathway [9,10]. Although PD pathology is now known to have many nonmotor manifestations due to involvement of other structures, the lesioned nigrostriatal pathway is a useful means of assessing the motor, dopaminergic responsive, aspects of the disease.

In the 1980s, it was shown by a number of labs, but most notably that of Anders Björklund, that the developing dopaminergic cells from the fVM could survive when grafted into the lateral ventricles, transplant cavity or as a cell suspension into the striatum of the lesioned rat [11–15]. These studies not only showed that the grafts survived well but that they also led to behavioral improvements, especially motorically. Further studies then went on to show that the dopaminergic cells in the graft were responsible for the behavioral recovery [16] and that they received and made synapses with the host brain [17]. On the basis of this work clinical trials with the tissue started in 1987 based on the primary principle of cell replacement. Other groups however, have sought to use cell-based therapies as a means to deliver trophic factors to rescue and support the remaining dopaminergic cells [18,19]. As of yet, the evidence that this can be used in a clinically meaningful way is still unproven.

However, even at the time when these initial clinical trials with fVM were being undertaken, there was recognition of the potential difficulties of using this tissue in terms of ethical and logistical concerns. Other catecholamine-producing tissues were therefore looked at, including adrenal medullary cells (which release a small amount of dopamine), with transplants also undertaken in lesioned rats [20]. However, these adrenal medullary grafts survived poorly, with at best modest improvements in motor deficits [21].

## First clinical trials

### Grafts involving cells other than fVM

Although the preclinical evidence for adrenal medullary graft function was poor, the first transplants in human trials were carried out using autologous adrenal medullary tissue. A number of patients received grafts using stereotaxic or open neurosurgical approaches, in several open-label trials [22–29]. However, results of these studies were highly variable, with clinical improvements generally being only modest or short-lived, and associated with a significant morbidity. Furthermore, postmortem analysis suggested that graft survival was poor [29], and thus this whole approach was abandoned in the early 1990s.

At this time there was also interest in the use of other dopamine-like cells including human retinal pigment epithelial cells, which have the capacity to produce levodopa and neurotrophic factors. After safety had been demonstrated in mouse and simian models, an open-label Phase I trial was conducted, in which postmortem human retinal pigment epithelial cells attached to microcarriers (Spheramine^®^ – Titan Pharmaceuticals Inc, MA, USA) were transplanted into the putamen of six patients with PD [30]. Though initial results were promising, with a sustained improvement in motor scores being reported, a subsequent randomized double-blind Phase II trial found that there was no treatment effect when compared with sham surgery controls [30,31] and at postmortem there was very poor graft survival [31]. Other approaches that have been trialed with limited success have included autologous carotid body cell grafts [32], and embryonic porcine mesencephalic tissue grafts [33], but all have given results which are significantly inferior to that seen with human fVM allografts (Box 1).

### Human fVM grafts

Following the promising preclinical transplant studies involving fVM tissue in animal models of PD, a series of small open-label studies in patients with PD was undertaken in Lund, Sweden. Though the first two patients that received such grafts did not experience clinical improvement, altering the transplant protocol (namely the amount of tissue transplanted, the use of tissue from fetuses of a slightly earlier gestational age and a smaller implantation needle) heralded improvement in motor features and ^18^F-DOPA PET imaging for the next two patients [34,35]. Eighteen patients in total in Lund were grafted, with an overall improvement in motor function in the majority of the patients, with some patients even managing to discontinue their PD medications, with long-term graft survival and clinical benefits [36,37].

One of the original Lund cohorts has recently come to postmortem, 24 years postgraft implantation, which has provided important insight into the long-term function of the fVM graft [8]. He initially achieved an excellent clinical response to grafting, and was able to come off levodopa completely for 3.5 years, and he was subsequently maintained on a significantly lower dose of levodopa (compared with the pregraft dose) for over a decade postgrafting. Fourteen years following implantation, the patient developed cognitive decline, and 4 years later no graft-related motor improvements persisted. At postmortem, the graft demonstrated robust survival with a dense dopaminergic reinnervation, though 11% of the neurons demonstrated Lewy body pathology [8]. The mechanism by which Lewy body pathology develops in the implanted cells is not known, and it may reflect transfer of α-synuclein pathology from the host to the graft, or it may occur as a result of inflammation at the graft site [38]. The presence of Lewy body pathology within the graft is a phenomenon that should not in itself limit the adoption of cell-based therapies – given that the majority of implanted neurons do not demonstrate such pathology, it is unlikely to cause graft dysfunction, at least for several years, during which time significant benefit can be seen [38]. The clinical outcome of this patient demonstrates that although neural grafting can alleviate motor symptoms and allow for reduced use of dopaminergic medications, as would be expected, the ongoing propagation of α-synucleinopathy to other areas of the brain and neurodegeneration may still ultimately herald clinical decline [8].

Encouraging results from these open-label Phase I trials led on to two double-blind trials in the USA, using sham surgery controls [5,6]. In these trials 43 patients with moderately advanced PD initially received human fVM grafts (with a further 13 of the sham surgery control patients from the first study receiving grafts after 1 year [5]). In the study by Freed *et al*., patients received grafts derived from the tissue of one fVM per side with no immunosuppression [5]. This compared with either one or four fVM transplants per side in the study by Olanow *et al*., which was followed by 6 months of cyclosporin [6]. Overall, both studies concluded that human fVM grafts did not provide significant improvement in patients with PD, with a high proportion of patients suffering graft-induced dyskinesias. These graft-induced dyskinesias typically developed in the first year, and persisted off medication. They were reported in 15 and 56% of the grafted patients in the Freed *et al*. and Olanow *et al*. studies, respectively [5,6]. In the first trial, development of graft-induced dyskinesia was associated with pre-existing levodopa-induced dyskinesia, and occurred in patients with severe fluctuations in motor symptoms prior to surgery [5]. In some cases the graft-induced dyskinesia was so severe that deep brain stimulation or further neurosurgery was necessary [37]. The basis for these graft-induced dyskinesias is still unresolved but may relate to contaminating serotoninergic neurons in the graft, and the uneven distribution of grafted dopamine cells across the transplant target site [39,40]. This high incidence of an adverse effect, coupled with a lack of efficacy in these trials, cast doubt over the whole approach.

## The TRANSEURO trial

Though the initial open-label trials suggested that most promise lay with human fVM grafts, the conclusions from the double-blinded American trials were disappointing. However, it was clear that some patients did achieve benefit from human fVM grafting, and indeed in the Olanow *et al*. trial, patients with less advanced PD achieved significant clinical improvements following grafting [6]. In addition there were a number of issues with the trials relating to patient selection, tissue preparation, trial end points and the level of immunosuppression adopted, which probably compromised the integrity and survival of the grafted tissue. Indeed the fact that the two trials adopted such different strategies clearly shows just how far the field was at this time from knowing how to optimally deliver such tissue – which is needed before double-blind trials are undertaken [37].

With the emergence of deep brain stimulation as a therapeutic option in PD, there was therefore a risk of the human fVM and other cell-based therapies being abandoned prematurely. A consortium was established, with a view to analyzing all of the available preclinical and clinical data, to review whether this whole approach had merit, and if so, to establish the optimal approach and identify which patients might gain the most benefit [37]. Earlier trials had utilized differing amounts of human fVM implants and immunosuppression, and had been conducted in patients at variable stages of PD. Positive outcomes were identified to be more likely in patients with less advanced PD, a younger age of onset with preserved ventral striatal dopaminergic innervation on ^18^F-DOPA PET, and preclinically in animals without pre-existing levodopa-induced dyskinesia [37,38]. Analysis also identified that the most effective grafts contained at least 100,000 dopaminergic nigral neurons (from three to four fetuses per side grafted), with a 12-month period at least of adequate immunosuppression postgrafting [37].

On the basis of the above analysis, a new open-label clinical trial was established (TRANSEURO), in which rigorous patient selection coupled with a refined approach to grafting has been employed (Table 1) [41]. As part of this trial, a small cohort of patients under the age of 65 with minimal levodopa-induced dyskinesias is receiving grafts containing tissue from at least three human fVM per side, followed thereafter with 12 months of standard triple immunotherapy. The primary end point will be the Unified Parkinson's Disease Rating Scale motor score in the defined ‘off’ state, 3 years postgrafting. The results of this study are expected to be published in 2020, with the hope that it will demonstrate that clinical improvement and safety can be consistently achieved with a more standardized approach for delivering a cell-based restoration of dopaminergic tone in the Parkinsonian putamen, paving the way for future clinical trials including those involving stem cell therapies.

## The stem cell era

Though the conclusion of TRANSEURO is awaited, the trials of human fVM grafting have suggested that in the appropriate population, cell-based dopamine replacement therapy can be done safely and could be effective. Though this has provided important proof-of-concept, the fact that at least three human fVMs are required per side grafted, coupled with the ethical implications of use of fetal tissue, means that human fVM therapies will never be useful for widespread clinical use. With evidence that dopamine-producing grafts can work in PD, the focus is now turning to identifying a means of delivering dopaminergic neurons in a clinically feasible manner, and there has been much interest in replicating the function of human fVM grafts using stem cell sources (Figure 1).

Dopaminergic neurons can be derived from human embryonic stem (ES) cells and this has become a promising prospect for cell replacement in PD. After the initial description of human ES cells, there was a great deal of interest in how these cells could be used as a source of specific cell types, for example, for disease-modeling and potentially transplantation medicine [42]. A variety of protocols were subsequently utilized with an aim of generating dopaminergic neurons that expressed tyrosine hydrolase (the rate-limiting enzyme in dopamine synthesis [37,43]). Though tyrosine hydrolase-expressing cells with the capacity to secrete dopamine were derived, these cells differed from true nigral dopaminergic neurons in that they neither express the key transcription factors LMX1A and FOXA2, nor develop the axons necessary to innervate the striatum, casting some doubt on how effective this approach could be [37]. The incomplete differentiation of these neurons also raised concerns regarding the potential for tumorigenesis, with teratoma-formation identified in some cases [43,44].

While there was ongoing interest in the use of ES cells as a source for cell replacement therapy, the induction of pluripotency in human fibroblasts in 2006 spawned a new avenue to explore [45]. These induced pluripotent stem cells (iPSCs) were hoped to be a source of patient-specific neurons, with a reduced chance of immune-mediated graft rejection. These cells responded in a similar manner to differentiation cues to ES cells, and dopamine-producing neurons were derived and grafted into animal models [46–48]. As with ES cells, there was uncertainty about the extent to which the iPSC-derived neurons reflected authentic nigral neurons [37], and in addition, issues of cost and safety through the reprogramming still exist [49].

The unexpected but key discovery that nigral dopaminergic neurons were in fact uniquely of floor plate (rather than neuroepithelial) origin, allowed for effective differentiation protocols to be developed, heralding the generation of genuine A9 dopaminergic nigral neurons that expressed floor plate markers [50]. This recognition of the floor plate origin of mesencephalic dopaminergic neurons was a crucial step toward the development of dopamine-producing neurons that could potentially be used in clinical trials. With this new insight, the differentiation process was refined, and dopamine-producing neurons could now be generated from both human ES cells and iPSCs with high efficiency, and increased graft survival and functionality compared with the neuroepithelial-patterned cells that had originally been described [51,52]. The derived dopaminergic neurons conveyed comparable function to fetal dopaminergic neurons [53], and the potential for tumorigenesis also seemed to have been circumvented, meaning stem cell-derived neurons had become the leading candidates for future clinical trials [37,51–52].

## Conclusion

Cell-based therapies for neurodegenerative disease have faced several challenges over the previous five decades. PD serves as the paradigm for the development of cell-based therapy in neurology, and neural-grafting techniques have evolved from relatively rudimentary adrenal medullary and fVM grafts, to the production of patient-specific, disease-specific grafts of subtype neurons, derived from a variety of stem cell sources. The nature of neural grafting has meant that progress has been stepwise and iterative, as processes have been refined and new knowledge has been gained. We are now on the brink of entering the clinical trial era for stem cell-based grafts, and though cell-based therapies are not yet ready for widespread clinical use, significant steps toward the clinic will be made over the coming decade.

## Future perspective

The progress that has been made over the previous three decades has brought us to the point where we now have the means to produce authentic nigral dopaminergic neurons from stem cell sources, which have potential to survive, innervate and function after grafting, without the problems of overgrowth and tumorigenesis [37,51–53]. Effective grafting for treatment of PD, therefore, seems feasible.

The first in-human trials of human ES cell-derived dopaminergic neuron grafts are on the horizon both in Europe and in the USA, with similar iPSC programs in Japan, and a global consortium (G-FORCE PD) has been established to ensure that stem cell therapies for PD progress toward the clinic safely and effectively [54]. In all these trials grafting is likely to begin within the next few years. In view of the uncertainties, ethical considerations and potential complications, any progress through in-human clinical trials will be iterative, as we try to establish the optimal dose of implanted stem cells needed for functional benefit, the best transplantation protocol and immunosuppressive regime and the optimal patient population and disease stage [55]. Initial trials will involve only a small number of patients, and grafts will likely be of a suboptimal number of neurons to minimize any risk of graft overgrowth [55]. Long-term follow-up will be required, as well as ultimately postmortem analysis of the grafts to determine rates of graft survival and also the development of Lewy body pathology within the grafted neurons. In-human Phase II trials are likely to be several years away, and it is important that studies are only carried out on the basis of robust preclinical and Phase I data, as unreliable conclusions from any premature studies would have the potential to derail progress in this field of stem cell-based regenerative therapies.

The derivation of neurons directly from adult somatic cells (e.g., fibroblasts), without passing through a stem cell intermediate stage, is an evolving field which may also herald a source of cell-based therapies in the future [56–58]. As with iPSCs, these cells could potentially provide a source of patient-specific neurons, and the absence of passage through a stem cell intermediate theoretically reduces the risk of tumorigenesis. Fibroblasts are directly converted to neurons by transducing differentiation factors, predominantly using lentivirus vectors [56–59]. One disadvantage of avoiding the stem cell stage, is that the number of neurons that can be generated will be finite, depending on the number of fibroblasts available. Initial protocols have required continuous supply of doxycycline, as the vectors have been regulated by a tetracycline-dependent transactivator. However, self-regulating vectors [59] have been developed and refined protocols have allowed for the efficient production of neurons – a crucial development if clinical trials are to be feasible using this approach. While these induced neurons hold promise, a consistent means of developing dopaminergic neurons from adult fibroblasts will need to be established before they can be used in clinical trials, and then whether it makes sense to use allogeneic as opposed to autologous tissue.

**Table 1.** **Details of the ongoing TRANSEURO trial.**

****Trial feature****	****TRANSEURO trial design****
Trial design	Open label
	Rater-blinded
Study arms	Interventional:
	– Allografting of at least three human fVM grafts per side
	– Graft tissue preparation consistent with GMP
	– Receiving 12 months of triple immunotherapy (cyclosporin A, azathioprine, prednisolone)
	Control:
	– No transplant or immunosuppression
	– Follow-up with the same clinical and imaging assessments as in the interventional arm
Grafting procedure	Rehncrona instrument used to introduce grafts to posterior putamen via five to seven tracts
Inclusion criteria	PD as defined using Queen's Square Brain Bank criteria
	Disease duration ≥2 years and ≤13 years
	Age ≥30 and ≤68 years at the time of grafting
	Hoehn & Yahr stage 2.5 or better in ‘on’ state
	On standard anti-PD medication without significant levodopa-induced dyskinesia (defined as a score of >2 on the AIMS dyskinesia rating scale, in any body part)
	Right handed
Selected exclusion criteria	Atypical or secondary parkinsonism (including F-DOPA PET appearance consistent with this)
	Clinically insignificant response to levodopa or apomorphine challenge
	Cognitive impairment (Mini-Mental State Examination score <26)
	Ongoing major medical or psychiatric disorder
	Concomitant treatment with neuroleptics and cholinesterase inhibitors
	Significant drug-induced dyskinesia in any body part
	Previous neurosurgery, cell therapy or organ transplantation
	Any contraindication to immunosuppression therapy
	Anticoagulation therapy
Primary end point	Change in motor UPDRS in the defined ‘Off’ state at 36 months postgrafting

AIMS: Abnormal Involuntary Movement Scale; F-DOPA: Fluorodopa ^18^F; fVM: Fetal ventral mesencephalon; GMP: good manufacturing practice; PD: Parkinson's disease; UPDRS: Unified Parkinson's Disease Rating Scale.

**Box 1.**  **Cell sources of dopamine replacement for Parkinson's disease that have been or are expected to be trialed in patients.**

**Autografts**

Adrenal medullary tissue
Catecholamine-producing tissue, which releases small amount of dopamine
Carotid body cells
Release a variety of mediators including glial cell line derived neurotropic factor and dopamine
Induced pluripotent stem cells
Derived from somatic cells such as fibroblasts, and converted into specific midbrain dopaminergic neurons
Induced neurons (yet to be investigated in patients)
Derived directly from somatic cells without a stem cell intermediate

**Allografts**

Fetal ventral mesencephalon
Containing neural progenitor cells, which differentiate into dopamine-producing neurons
Retinal pigment epithelium/Spheramine^®^
Harvested from postmortem human eyes, produces levodopa and growth factors, and linked to specific microcarriers for transplantation
Embryonic stem cells
Harvested from preimplantation embryo, and differentiated into subtype neurons including dopaminergic neurons

**Xenografts**

Embryonic porcine mesencephalic tissue
Containing developing porcine dopaminergic neurons

**Executive summary**

**Challenges for cell-based therapies**

Cell-based therapies pose an array of challenges including ethical considerations, immune-mediated rejection and risk of tumorigenesis and graft overgrowth, which have had to be overcome in order to progress toward clinically useful treatments.
In addition, they have to compete with a range of very effective therapies that target the same dysfunctional dopaminergic network in Parkinson's disease (PD) – thus questions over their ultimate competitiveness in the clinic exists.

**Preclinical studies**

Several studies in animal models of PD were initially undertaken using fetal ventral mesencephalon (fVM) and adrenal medullary grafts.
fVM grafts demonstrated good survival and behavioral improvements in animal models of PD, whereas adrenal medullary and retinal pigment epithelial grafts demonstrated poor survival and function, which was then seen clinically showing that these models are predictive of clinical efficacy.

**First clinical trials**

An array of cell sources have been used in clinical trials, including adrenal medullary tissue, human fVM, retinal pigment epithelial, carotid body cells and embryonic porcine mesencephalon tissue.
Human fVM grafts appeared to be the most promising approach, and though initial studies reported variable results with significant adverse effects (including two double-blinded trials involving sham surgery controls), some patients clearly derived marked long-term benefit.

**The TRANSEURO trial**

The TRANSEURO trial was established after detailed analysis of all of the available data from preceding human fVM transplant trials, culminating in a refined approach to patient selection, graft preparation and implantation, immunosuppression and trial design. Results are awaited.

**The stem cell era & future perspective**

The discovery that nigral dopaminergic neurons were derived from the floor plate allowed for the effective production of authentic A9 nigral dopaminergic neurons from embryonic stem cells and induced pluripotent stem cells, potentially providing a patient- and disease-specific population of cells for grafting.
Embryonic stem cells appear to be the most promising source of a cell-based treatment for widespread clinical use, with the first in-human clinical trial of embryonic stem cell grafts for PD on the horizon.
There is ongoing interest in the direct conversion of adult somatic cells (e.g. fibroblasts) to neurons (induced neurons), which may provide an alternative avenue for cell-based therapies in the future.
